# Effects of modes of climate variability on wave power during boreal summer in the western North Pacific

**DOI:** 10.1038/s41598-020-62138-0

**Published:** 2020-03-20

**Authors:** Sinil Yang, Jai-Ho Oh

**Affiliations:** 1 Korea Ocean Satellite Center, Korea Institute of Ocean Science and Technology, Busan, 49111 Republic of Korea; 2 Department of Environmental Atmospheric Sciences, Pukyong National University, Busan, 48513 Republic of Korea

**Keywords:** Atmospheric science, Ocean sciences

## Abstract

This study investigates extremes of wave climate in the western North Pacific (WNP) as significant responses to modes of climate variability: the El Niño-Southern Oscillation (ENSO) and the Pacific Decadal Oscillation (PDO). Few studies have explicitly investigated significant wave height in this context, and hence, the aim of the present study is to investigate and quantify the responses to a simulated wave data set over the WNP from 1979–2009 by focusing on the combined influences of the ENSO and PDO during the boreal summer. We conducted a composite analysis of sea surface temperatures, sea-level pressure, and extreme anomalies of wave power density (Pw) on different phase combinations of the ENSO and the PDO, and also analyzed the effects of a latitudinal shift of the ITCZ for composite samples with respect to simulated tropical storm (TS) activities. The results demonstrate that the ENSO played a primarily positive role in intensifying anomalous wave climate, while the PDO had the opposite effect. The responses of the peak wave-period were linked to a strengthened anomalous low-pressure and a cooling of sea surface temperature anomalies. The PDO played a significant role in strengthening or weakening of the effects of the ENSO on Pw, thus confirming the findings of previous studies. We found that responses were dependent on whether ENSO and PDO were in or out of phase. These responses can be described by a strengthening of the southeast trade winds that blow across the equator with respect to a latitudinal shift of the Intertropical Convergence Zone (ITCZ). Our findings contribute to the understanding of a relationship between modes of climate variability and TS activities with respect to the status of the ITCZ over the WNP, which can be relevant factors in the lifetime of wave power and related wave parameters in the WNP during the boreal summer.

## Introduction

Extreme wave climates are crucial to the development of various natural processes^1^. It is essential to forecast and reproduce regional wave characteristics in the ocean. Additionally, the ocean wave has the highest energy density in coastal marine areas^2^. Thus, considerable research on the regional wave climate has been conducted to evaluate and characterize the wave power energy potential. Specifically, and with reference to climate variability, previous studies have attempted to improve the understanding between significant wave height (hereafter Hs) and seasonal oscillations on global^3–5^ and regional scales^1,6–11^, and for future projections^12,13^. The effects of long-term wave climate variability in the western North Pacific (WNP) have also been reported^14,15^. Sasaki (2012)^14^ evaluated the regional wave climate based on 30 years of observational data and identified an significant positive trend that was induced by more frequent swells. Allan and Komar (2000)^15^ reported an increasing Hs trend over the eastern North Pacific (ENP) over approximately 25 years previous to their study, which may have been linked to the El Niño-Southern Oscillation (ENSO). Additionally, because the ENSO modulates tropical storm (TS) activities in the North Pacific^16,17^, TS activities in the WNP− in addition to the ENSO− also have a substantial influence on the wave climate. Previous studies^1,18,19^ have demonstrated that the number of TS could be essential for affecting variations in the Hs in the WNP during the boreal summer, which is linked to warm sea surface temperature (SST) anomalies in the Niño 3.4 region [5°S–5°N, 170°–120°W]. However, few studies have explicitly investigated wave power density (hereafter Pw), which is defined by a function of the Hs and peak wave period (hereafter Tp)^20,21^. A longer Tp produces a greater wave run-up, which raises flooding potential^6,22^. Therefore, Pw is more useful than Hs alone for describing the magnitude of storm effects, which depends on wave energy brought by long waves^23,24^.

However, previous studies^9,25^ did not address the role of the intertropical convergence zone (ITCZ) and trade winds on extremes of Pw in the WNP during JJA. Consistent trade winds (e.g., northeasterly and southeasterly) are among the tropical climate phenomena. The trade winds regularly blow towards the equator, where extreme precipitation occurs in the ITCZ. However, the ITCZ varies by seasonal latitudinal shifts. Richter *et al*.^26^ explained that the ITCZ moves approximately 8°N from its southernmost latitude during the boreal spring to its northernmost latitude during the boreal summer, which is caused by the strengthening of the southeast trade winds (i.e., the equatorial easterlies) blowing across the equator from the southern hemisphere. Previous studies^27,28^ have demonstrated that the latitudinal shift of the ITCZ affects SST warming to the north of the equator. Furthermore, Vincent *et al*.^29^ suggested that the influence of the ENSO along the ITCZ appears to be associated with intensified TS activities during El-Niño. In particular, the wind variability in the WNP is dominated by subtropical, large-scale atmospheric circulation and affects the regional wave climate during the boreal summer (i.e., June, July, and August (JJA)). For instance, Tao *et al*.^30^ revealed that the tropical, large-scale atmospheric circulation was accompanied by the development of anomalous cyclones in the WNP. Furthermore, Zhang *et al*.^31^ found a relationship between the central Pacific (CP) cooling and the westerlies (i.e., the Rossby wave) response to the part of the northwestern CP^32^.

The aim of the present study is to investigate and quantify the mean distribution and extreme trends of a simulated wave data set over the WNP from 1979–2009, by mainly focusing on the combined influences of the ENSO and PDO during the boreal summer. To investigate influencing factors, we conducted a composite analysis of SST, sea-level pressure (SLP), and extreme anomalies of Pw on different phase combinations of the ENSO and PDO. In particular, we analyze the effects of a latitudinal shift of the ITCZ for each composite sample with respect to TS activities.

## Results

### Influence of modes of climate variability

#### SST and SLP

**Figure 1:**
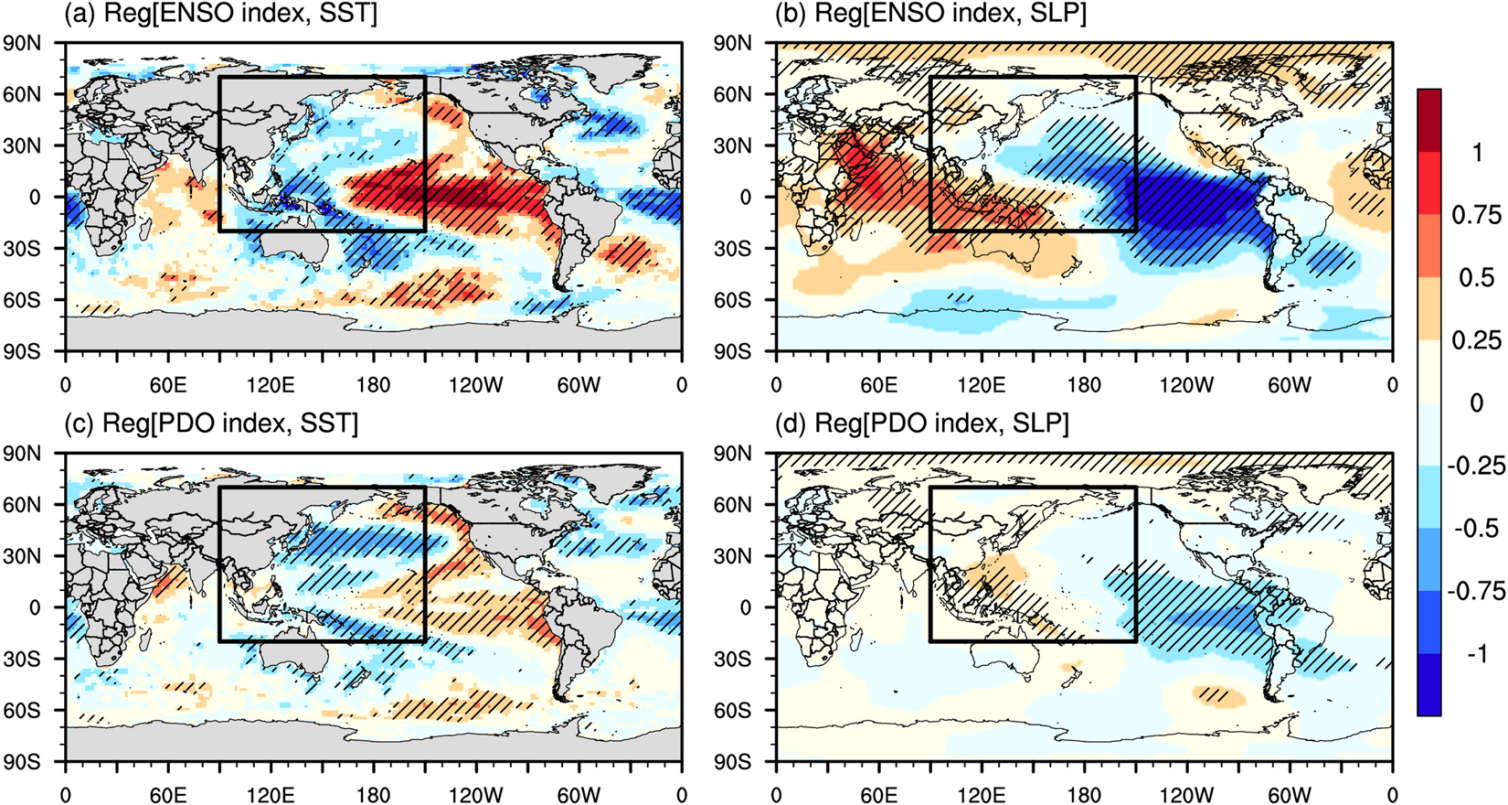
Linear regression spatial patterns of standardized sea surface temperature (SST, left) and sea-level pressure (SLP, right) onto (**a**,**b**) the El Niño-Southern Oscillation (ENSO), and (**c**,**d**) the Pacific Decadal Oscillation (PDO) indices during June, July, and August (JJA) from 1979–2009. The analysis domain is shown as a black box covering [20°S–70°N, 90°E–150°W]. The hatching represents grid points with significant regression coefficients at the 5% level.

 Figure 1 illustrates the influence of each mode of climate variability on both SST and SLP as defined by linear regression analysis. The ENSO can be seen as a principal mode among the modes of climate variability for both SST and SLP in the tropical Pacific (Fig. 1a,b). The most significant relationship (p < 0.05) between the ENSO and SSTs with a regression slope larger than 0.5 was found over the eastern Tropical Pacific (Fig. 1a). Furthermore, the effects of the ENSO were found to reach far beyond the tropical Pacific, consistent with the findings of Hu *et al*.^33^. In particular, statistically significant (hereafter at the 5% level) responses of the ENSO to SSTs were recognized from the CP to the eastern Pacific (EP) and the tropical Indian Oceans, where warm conditions prevail, whereas cold conditions prevail in the western tropical Pacific. Additionally, we found a significant relationship (p < 0.05) between the PDO and SSTs with a positive sign in the mid-Pacific Ocean and a negative sign in the WNP (Fig. 1c). Furthermore, the ENSO and the PDO had a substantial impact on the SLP in the anomalous low-pressure region of the northeastern Pacific, and the anomalous high-pressure regions in the CP and Indian Oceans (Fig. 1b,d). Thus, anomalous pressure with a sustained wind speed could lead to the 99^*th*^ percentile of wave parameters: Hs_99_ and Pw_99_. The impact of the PDO was found to be related to SST warming over the northeastern North Pacific, SST cooling over the northwestern North Pacific, and anomalous low-pressure over the central and northeastern North Pacific (Fig. 1c,d).

#### Extremes of wave climate

**Figure 2:**
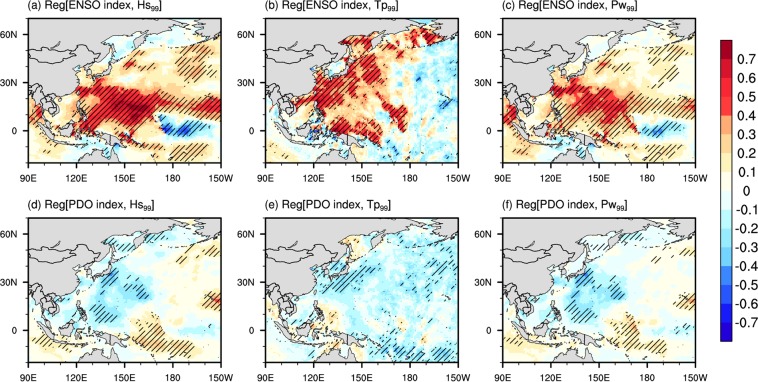
Linear regression spatial patterns of standardized 99^*th*^ percentile of wave parameters: significant wave height (Hs_99_, left), peak wave period (Tp_99_, center), and wave power density (Pw_99_, right) onto (**a**–**c**) the El Niño-Southern Oscillation (ENSO), and (**d**–**f**) the Pacific Decadal Oscillation (PDO) indices during June, July, and August (JJA) from 1979–2009. The hatching represents grid points with significant regression coefficients at the 5% level.

 Figure 2 presents the spatial regression maps for extremes of the wave climate, which were regressed onto the modes of climate variability. The warm ENSO phase typically exhibited a positive response to wave parameters in the WNP, which was contrary to the influence of the PDO. The influence of the warm ENSO phase on the Pw was statistically significant, and was found to be mainly a positive response in the WNP and a negative response in the CP (Fig. 2c). The strongest statistically significant responses of the Pw_99_ were located in the WNP along the 20°N latitude. The positive influences of the ENSO along the region seem to be correlated with enhanced TS activities during El Niño. This is because increased, sustained wind-speed was a dominant factor causing increased Pw_99_, which is consistent with the findings of Yang and Oh (2017)^1^. The response of the PDO to the Pw was approximately opposite to the ENSO (Fig. 2c,f), but few responses were significant at the 5% level. Finally, for the positive PDO phase, the regions that exhibited negative responses were located in the WNP, which spans the western coast of the WNP to the mid-Pacific and relates to the storm tracks that are developed in the mid-latitudes of the WNP. The positive PDO phase had a significant negative impact on the Pw over the WNP, which covers almost the same region as that influences by the ENSO; hence, there was an inverse relationship (i.e., out of phase) to the ENSO influence in the WNP (Fig. 2a,d).

Additionally, the statistically significant areas (p < 0.05) of the ENSO and the PDO influences on the Hs_99_ were mainly similar to the response of the Pw_99_ because the Hs is a dominant factor when calculating the Pw in Eq. (1) (see Methods). Figure 2a presents a positive response of the Hs_99_ over an area that spans from the WNP to the tropical Pacific along 20°N latitudes, and the impact of changes in SST anomalies in the ENP. Contrary to the Hs_99_, the response of the warm ENSO phase on the Tp_99_ was higher in the WNP and lower in the central North Pacific (Fig. 2b). Most of the positive responses to the warm ENSO phase were found to exist along the western basin of the WNP (e.g., the East China Sea and the Philippine Sea). The notable regions imply a substantial relationship, whereby changes to tropical SST anomalies due to the warm ENSO phase resulted in variations in anomalous long Tp_99_. On the other hand, the response to the positive PDO phase was nearly negative across the WNP and of lesser significance to that of the warm ENSO phase at the 5% level, which was contrary to the ENSO response (Fig. 2b,e). Overall, the northwestern WNP region with a significant response of the Tp_99_ to the ENSO was correlated with the strengthening of the anomalous low-pressure (high-pressure) and cooling (warming) of the WNP in in-phase (out-of-phase) between ENSO and PDO.

### Composite analysis

The aforementioned analysis of the effects of the chosen climate indices on the Pw was achieved by applying the influence of individual climate indices to each other. To further investigate the combined influences of the dominant climate mode in the WNP, we conducted a composite analysis of the SST, SLP, and Pw_99_ anomalies on different phase combinations of the ENSO and the PDO. In particular, our analyses aimed to determine the effects of TS activities near the ITCZ between the ENSO and the trade wind index (TWI).

#### Influence of ENSO and PDO

**Figure 3:**
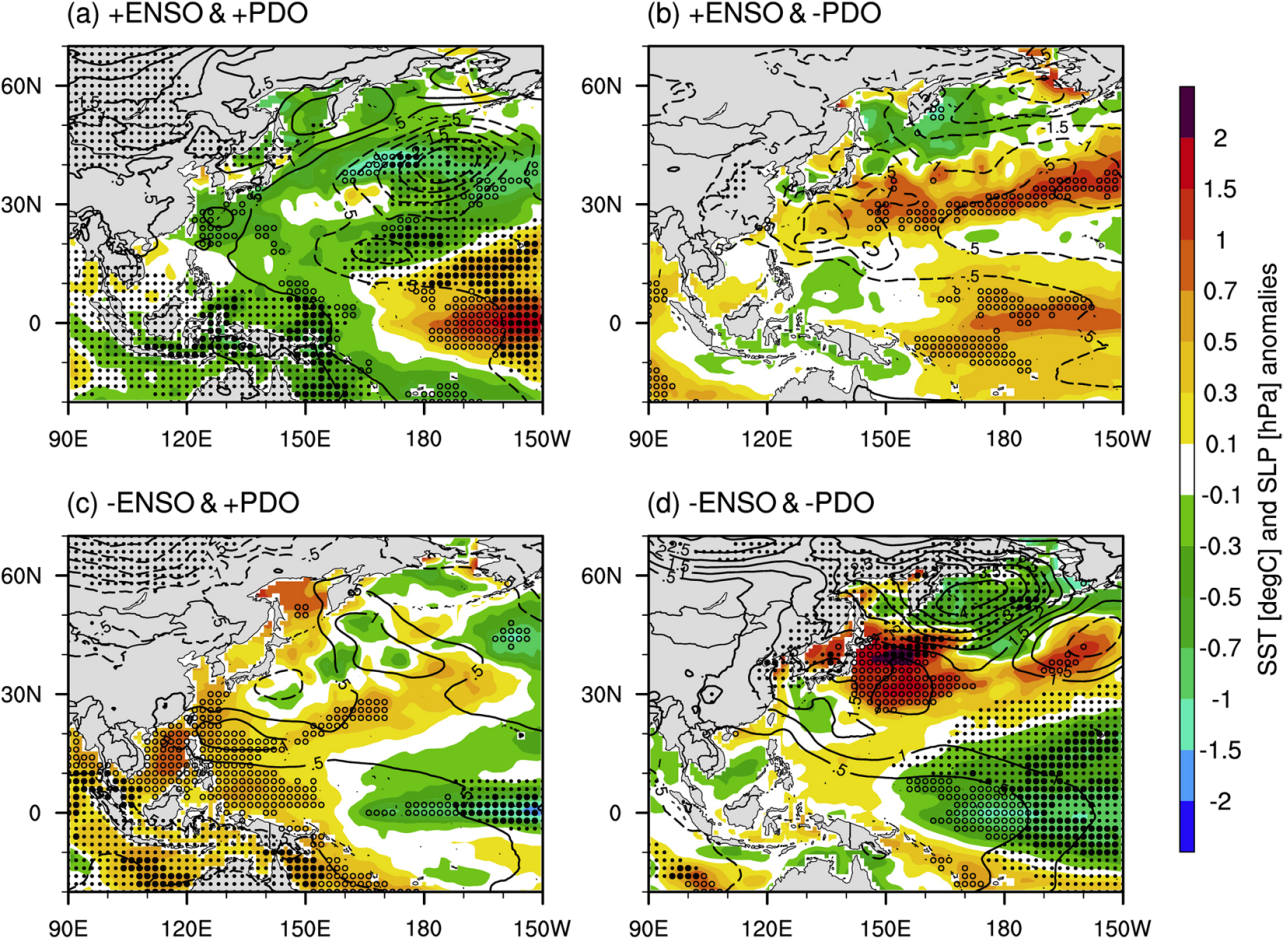
Composite patterns of sea surface temperature (SST, shaded area, in degrees Celsius] and sea-level pressure (SLP, contours, in hPa) anomalies for the four combinations of the El Niño-Southern Oscillation (ENSO) and the Pacific Decadal Oscillation (PDO), which are the (**a**) El Niño (+ENSO)/+PDO, (**b**) +ENSO/−PDO, (**c**) La Niña (−ENSO)/+PDO, and (**d**) −ENSO/−PDO years. All anomalies are computed relative to the 31-year period from 1979–2009. Filled and unfilled dots denote significant SLP and SST anomalies at a confidence level of 90%, respectively.

**Figure 4:**
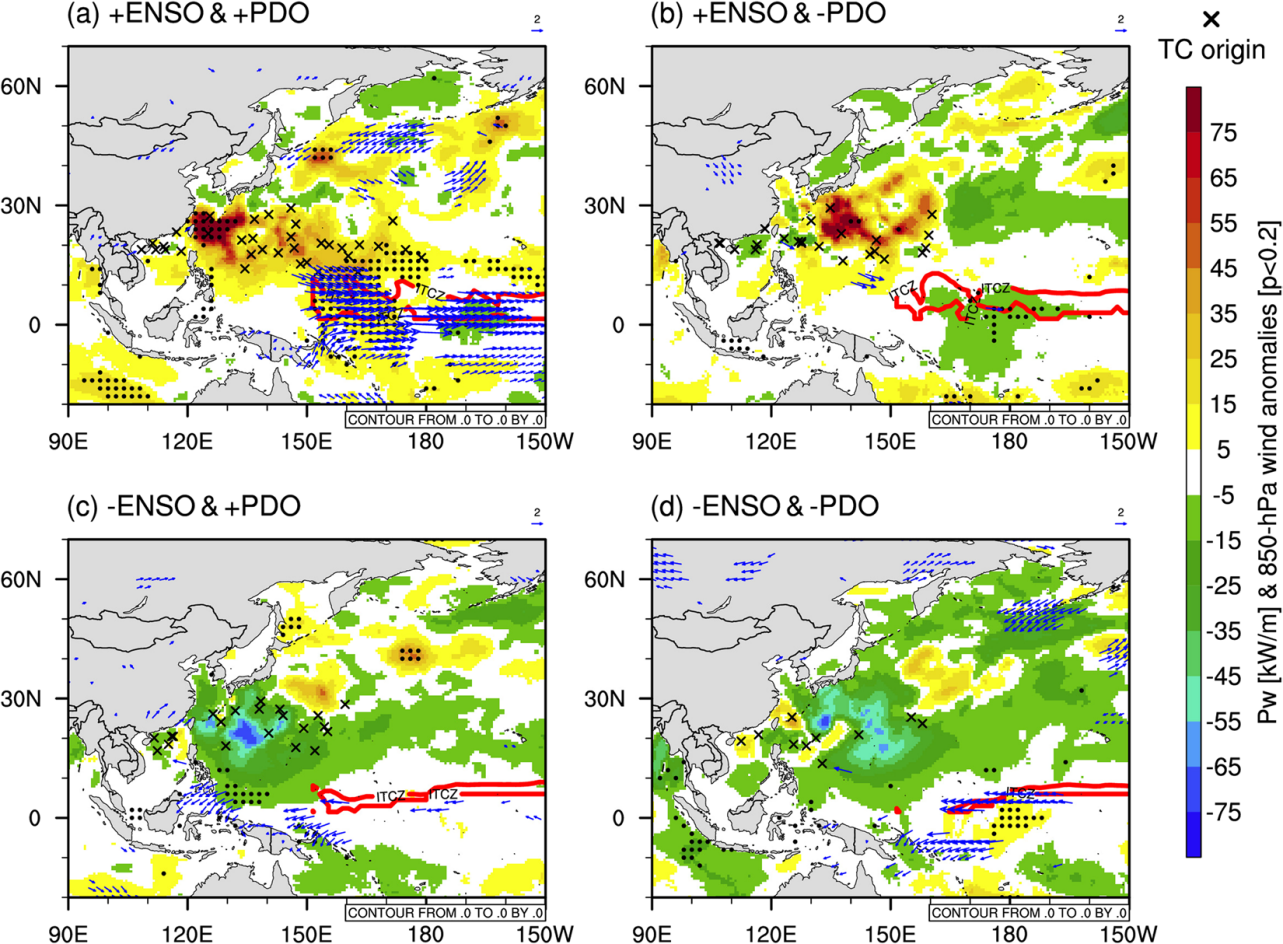
Composite patterns of Pw_99_ [shaded, in kW/m] anomalies for the four combinations of the El Niño-Southern Oscillation (ENSO) and the Pacific Decadal Oscillation (PDO), which are the (**a**) El Niño (+ENSO)/+PDO, (**b**) +ENSO/−PDO, (**c**) La Niña (−ENSO)/+PDO, and (**d**) −ENSO/−PDO years. The blue arrows indicate the significant wind vector anomalies at 850 hPa (vectors, in m/s) at a confidence level of 80%. All anomalies are computed relative to the 31-year period from 1979–2009. The black cross and solid red line represent the TS genesis location and the ITCZ area detected by the ICON model for each case, respectively. The black dot denotes the significant Pw anomalies at a confidence level of 90%.

We selected four composites from El Niño (hereafter +ENSO) and La Niña (hereafter  − ENSO) events based on the Ocean Niño Index (ONI) indices, and positive and negative phases (+PDO and  − PDO) of the PDO for comparison to the ±0.5-standard deviation. Table 1 shows that the classified sample year was 3, 2, 3, and 2 for each of the different phase combinations. Figures 3 and 4 show the SST, SLP, and Pw_99_ anomalies for the four composites of both the ENSO and the PDO. El Niño and La Niña events have been found to present typical SST anomalies in the equatorial tropical Pacific^34,35^. SST anomalies in the CP and EP are significantly warming during the +ENSO/+PDO (Fig. 3a) and significantly cooling during the −ENSO/−PDO (Fig. 3d). These results indicate strengthening of the ENSO impact when the PDO is in phase with the ENSO. When the ENSO and PDO are out of phase, we found that the spatial patterns of SST were described by the weakening of the ENSO impact, mainly over the CP (Fig. 3b,c). In terms of the influence on SLP, similarly to SST, anomalously low SLPs exist during +ENSO/+PDO and anomalously high SLPs exist during  − ENSO/ − PDO over the mid-central North Pacific in comparison to years with other phases (Fig. 3a,d). The results were clearly significant and confirmed that the PDO presents a substantial role in strengthening or weakening the ENSO effects on SST and SLP over the North Pacific, as previously reported^9,36^. Furthermore, Pw_99_ anomalies were found to increase during +ENSO/+PDO and decrease during −ENSO/− PDO around the WNP (Fig. 4). In this region, an +ENSO (−ENSO) related to +PDO (−PDO) was observed to cause substantial significant increases (non-significant decreases) in Pw_99_ anomalies. The SST in the central North Pacific was significantly cooling for +ENSO/+PDO events and significantly warming for +ENSO/−PDO events (Fig. 4a,b). These results indicate that the −PDO strengthening, when combined with +ENSO events, affected the sign and peak region of SST anomalies in the CP. Additionally, the significant intensity of the SST and SLP anomalies in the WNP also depends on the phase of the PDO for −ENSO/+PDO and −ENSO/−PDO events (Fig. 3c,d). In the case of −ENSO years, the Pw_99_ anomalies in the WNP also represented a negative sign regardless of the PDO phase, thus indicating that the ENSO played a substantial role in strengthening or weakening the Pw_99_ in the WNP (Fig. 4c,d). However, the ENSO had the most significant influence on the Pw_99_ during +ENSO/+PDO events (Fig. 4a), Pw_99_ anomalies during +ENSO/−PDO events were mostly non-significant in the WNP (Fig. 4b). This relationship means that the ENSO did not have a direct impact on the Pw_99_ in the WNP, rather an indirect impact. We observed that this indirect impact may be associated with the zonal trade wind and enhanced TS activity, especially during the boreal summer, along with the ITCZ. This relationship is analyzed in the following section.

**Table 1:** Classification and statistics for tropical storm (TS) activities of El Niño (+ENSO) or La Niña (−ENSO) and/or positive or negative PDO (+PDO or −PDO) year combinations > ±1.5 standard deviation occurring in June, July and August (JJA) from 1979–2009.

Composites	Sample years	Mean TWI	Number of TS	Mean origin location
+ENSO/+PDO	1982, 1987, and 1997	1.30	34	20. 2°N, 142. 1°E
+ENSO/−PDO	1991 and 2002	0.79	23	21. 0°N, 133. 7°E
−ENSO/+PDO	1988, 1998, and 2007	−0.99	21	22. 8°N, 136. 3°E
−ENSO/−PDO	1999 and 2000	−1.25	10	20. 6°N, 132. 8°E

The mean trade wind index (TWI) is the average of the normalized TWI for each case. The number of TS is the sum of the number of TS for all years in each composite. The mean origin location is the averaged location for all TS genesis in each composite.

#### Influence of ENSO and TS activities

The influences of the ENSO along the WNP region appear to be correlated with enhanced zonal trade winds because increased zonal wind-speed energy was a dominant factor in inducing changes in the Pw (see Fig. 2a,c). The positive influences of the ENSO, along with the ITCZ, seem to be related to TS activities, thus indicating that increased surface wind-speed energy indeed induced an increased Pw_99_ in the WNP. Forecasting properties of TS are important for gaining a better understanding of the modes of climate variability. There might be uncertainty because different analyses have used different data assimilation schemes and global climate models (GCMs)^37–39^ presented the impacts of ENSO on TS activity using climate models. There might be uncertainty because different analysis uses a different data assimilation scheme and global climate models (GCMs), respectively^40^. The Icosahedral Nonhydrostatic model (ICON) model has been verified by several idealized simulations, and has been used for the operational production of the German Weather Service (DWD) since 2015^41^. The use of a simulated wave data set of the type used by the Atmospheric Model Intercomparison Project (AMIP) to detect TS presents an opportunity to reduce the uncertainty by supporting prescribed SSTs and sea ice concentrations (SICs) when simulating the atmospheric results of the ICON. In particular, the ITCZ movement varies depending on the phases of ENSO and PDO. Thus, the geographical findings of the ITCZ can be important in interpreting the relationship between the waves and the modes of climate variability. Therefore, we have employed the objective identification of the ITCZ in Berry and Reeder (2014)^42^. To identify the ITCZ from numerical simulation, we computed the mean divergence between 1000 and 850 layers, the gradient of the divergence, and the wet-bulb potential temperature at 850 hPa, which are obtained from the ICON model. In the present study, we apply the same thresholds of the original method^42^ except the Laplacian of the divergence. Through this objective method, we have provided a simulated ITCZ area in each phase between ENSO and PDO. The ITCZ area represents solid red lines in each panel in the revised Figure 4.

The AMIP-typed simulation of the ICON can help to bring more confidence to TS detection used in the present study. Thus, we detected simulated TS (ranging from tropical storms to typhoons) in the ICON model using the TC tracking method of Murakami and Sugi (2010)^43^. From the composites of Fig. 4, we investigated the relationship between the Pw_99_ and SST anomalies with TS activities in JJA (Fig. 4). Over the tropical Pacific, the zonal SST gradient between cold water in the eastern part and warm water in the western part, was considered to be the cause of this thermally induced direct circulation, which is called the Walker circulation^44^. The normalized zonal wind index at 850 hPa, TWI, was used to describe the behavior of the near-surface wind over the WNP under the Walker circulation along the equator. Table 1 shows the number and mean origin location of TS for each of the different phase combinations, and the TWI. During El Niño years, the westerly zonal trade winds were strong and persistent and the mean TWI was  ~ 1.3. The genesis location of the TS shifted eastward across 150°E in the WNP during El Niño years in comparison to La Niña years (Fig. 4a–d). More TS occurred during an El Niño event in the eastern region of the WNP (150°–180°E) than in the western region (120°–150°E). The eastward shift in genesis locations was more pronounced during El Niño events, and most of the formation origin locations were found as far east as 110°–180° E along the 20°N latitude (Fig. 4a). Additionally, we found that TCs have a better chance of interacting with synoptic systems in the midlatitudes because of their longer lifespans. The finding is consistent with the results of Chu (2004)^45^, where the eastward and equatorward shift in genesis locations allowed the TS to survive longer during their movement toward the East Asian continent. Most of the origin locations of TS for La Niña events were confined to the west of 15°E during the summer (Table 1). The genesis locations during the summer remained at the midlatitudes (20°–30°N) in the WNP, which was due to the easterly trade winds toward the western Pacific where there are warm pools^46^. In particular, Figure S2 shows the composite means of Tp anomalies and its significance at a confidence level of 90% (see Supplementary Fig. S2). The ENSO and PDO contribute to a magnitude of Tp of tropical waves caused by trade winds blowing from the southern hemisphere. This mechanism is related to the propagation of large swell waves from near the equator to the northern region of the WNP (Fig. S2). This figure helps us understand the wave climate of the North Pacific that includes wind-driven waves and swells. Therefore, substantial variations in the Pw extremes were affected by TS caused by tropical SST anomalies and anomalous zonal trade winds (Fig. 4). The status of the ITCZ could be relevant to the anomalies of the Pw_99_ in the WNP, which is associated with atmospheric circulations in the tropical Pacific.

## Discussion

In summary, several dominant features (e.g., tropical storms, trade winds, monsoons, and modes of climate variability) contribute to the wave climate. The contributions include local wind waves affected by ITCZ movement, swells generated near the equator, and tropical storm activities. Most extreme waves, however, may be related to TS activities, though large swells may also cause some from ITCZ. Several studies have confirmed that the PDO plays an important role in the impact of ENSO on wave climate^9,36^. These findings imply that ENSO may be the sole source for modulating the effects of low-level wind on the wave climate near ITCZ. This ITCZ movement depends on the different phases of ENSO and PDO.

Extremes of the wave climate in the WNP exhibited considerable responses to the ENSO, the PDO, and the northward migration of the ITCZ. The ENSO was found to be the dominant mode of climate variability both in terms of SST and SLP in the tropical Pacific. In particular, the statistically significant areas for the influences of the ENSO and the PDO on the Hs_99_ were found to be mainly similar to the response on the Pw_99_, which is because the Hs is a dominant factor when calculating the Pw. The notable regions of Tp imply the significant relationship, whereby changes to tropical SST anomalies due to the ENSO result in substantial variations in anomalously long Tp_99_. On the other hand, the response of the Tp to the PDO is almost negative across the WNP and less significant to that of the ENSO. Overall, the statistically significant responses of the Tp_99_ were linked to a strengthening of the anomalous low-pressure in the WNP and the cooling of the WNP. In particular, the extremes of the wave parameters in the WNP could occur as either the warming and cooling of SST or the anomalous high and low-pressure accompanied by a sustained wind. Additionally, we found that extreme wave climates were a response to whether the ENSO and PDO were in phase or out of phase. Thus, our results confirmed that the PDO plays a statistically significant role in strengthening or weakening the ENSO influences on the Pw_99_ in the WNP, as previously reported^9,36^. Furthermore, Camargo *et al*.^47^ found that SST anomalies in the tropical ocean that were induced by ENSO could alter the atmospheric conditions in the WNP (e.g., atmospheric stability, zonal trade wind, and vertical wind shear). Since the ENSO modulates TS activities in the North Pacific^16,17^, the TS activities in the WNP, as well as the ENSO, have a substantial impact on the wave climate. Previous studies^1,18,19^ have shown that the number of TS created could be essential for affecting the variation of the Hs_99_ in the WNP during the boreal summer, which was linked to warm SST anomalies in the Niño 3.4 region.

In addition to the relevant factors, as mentioned above (e.g., ITCZ movement and southeast trade wind), several studies stated changes in SST, monsoon, and low-level relative vorticity contributed to changes in the frequency of TSs in the WNP during ENSO events^48–50^. This change implies that the TS activities can contribute to the wave climate in the WNP. For example, the positive influence of the ENSO appears to be related to enhanced TS activities during El Niño, and indicate that increased wind-speed energy induces increased extremes of Pw, as a previous study suggested^29^. In particular, Chan *et al*.^51^ examined the changes in the early summer monsoonal circulation over the South China Sea can be described as the change in the extension of the 500-hPa subtropical high depending on the ENSO coupled with the PDO. The variability of the monsoon trough can be also one of the relevant factors that influence the wave climate in the region of the WNP (e.g., the South China Sea). Further research may be needed in order to investigate the impact on the wave climate by modulating the monsoonal circulation. Similarly, combined effects of extreme sea levels due to the contribution of waves, tides, and pressure systems lead to extreme events (e.g. coastal inundation)^52^. The main causes of storm surge in the coastal region of the Pacific are from tropical cyclones (TCs), and several studies have investigated how they might change^53^. For example, the results of Wang and Zhou (2016)^52^ found the relationship between the important trends in extreme sea levels and the influence of tropical cyclones in Macau and Hong Kong. Thus, this relationship between ENSO and TCs will help us understand the changes in sea-level height and associated wave parameters for future projection in the WNP.

From the composite analysis in this study, we found that the SST and SLP anomalies related to the positive phase of the ENSO and PDO could be relevant to anomalies of extreme wave climate in the WNP. Also, the changes in the wave climate were related to the conditions of equatorial SST and low-level wind fields over the tropical Pacific as well as the TS activities. The response can be explained by a strengthening of the southeast trade winds (i.e., the equatorial easterlies) blowing across the equator in the southern hemisphere with respect to a northward shift of the ITCZ. These Northward shifts of the ITCZ can significantly increase Pw anomalies by increasing Tp anomalies in the WNP, i.e., the ITCZ contributes to the formation of large swells that propagate from the equator to the North Pacific. Thus, the present study contributes to the understanding of the (i) effects of the ENSO and the PDO on extreme wave climate, and (ii) relationship between the ENSO, the PDO, and TS activities with respect to latitudinal shifts of the ITCZ over the WNP. Therefore, the present findings can be important in interpreting the relationship between waves and modes of climate variability. The present study also indicates that the status of the ITCZ in the positive phase of ENSO and PDO can be a relevant factor in the lifetime of extreme wave climates in the WNP during the boreal summer.

## Data and Methods

### Data

#### Global wave data set

Following Yang and Oh (2017)^1^, the present study used the same simulation output in terms of Hs and Tp as a wave parameters. The global wave data set is available from January 1979 to December 2009 on regular latitude-longitude grids at 0.25° × 0.25° resolution at three-hour intervals. The gridded wave data set provides commonly used wave parameters applicable to the Hs, mean wave height, Tp, mean wave period, mean and peak wave direction, and wind sea wave period and direction. Yang and Oh (2017)^1^ used the full-spectral third-generation numerical wave model WAVEWATCH-III^54^. Their study employed the model to reproduce the global wave characteristics with the AMIP-typed hindcast from an atmospheric general circulation model (AGCM); ICON. The ICON model is a joint development project of the Max Planck Institute for Meteorology (MPI-M) and the DWD^41^. A detailed description of the ICON can be found as Supplementary text S1 online. Based on a procedure defined elsewhere^55^, the AMIP-type simulation was run with the prescribed SSTs and SICs observed by the National Center for Atmospheric Research (NCAR) to avoid an unrealistic condition of both SSTs and SICs. The Hs among the wave parameters was validated by a 30-year wave hindcast of Climate Forecast System Reanalysis and Reforecast (CFSRR)^56^, in which the area-weighted mean root mean squared error (RMSE) and correlation coefficient for the WNP (100°E–180°E, 0°–60°N) were 0.5 m and 0.69, respectively^1^. This CFSR hindcast provides three hourly global data with a spatial resolution of 0.5°, which is considered a reliable dataset among global hindcasts. Additionally, validation of the simulation results for Hs and Tp was also in agreement with the CFSR hindcast (see Supplementary text S2 and Fig. S1). The simulation results, therefore, are useful in the analysis domain and allows us to employ climate research.

#### Ancillary data set

Interannual climate variability in the WNP is partially induced by the behavior and strength of ocean-atmospheric oscillations. We used two climate indices: the PDO and the ENSO, which have 20–30 year cycles^57^ and 2–7 year periods^58^. Active phases of the PDO and ENSO have brought warmer and drier winters to the WNP region, while negative aspects have brought colder and wetter conditions^59,60^. Therefore, a couple of climate indices, the ONI and the PDO, were used to describe the climate variability of the ENSO and the PDO, respectively. To evaluate the relationship between the simulated wave climate and global climate variability, we obtained the following climate indices during JJA: (i) the ONI was obtained from the United States National Weather Service Climate Prediction Center (https://www.cpc.ncep.noaa.gov/data/indices/), derived as the three-month running average of SST anomalies over the Niño 3.4 region covering [5°S–5°N, 170°–120°W], and (ii) the PDO index was downloaded from the Joint Institute for the Study of the Atmosphere and Ocean (http://research.jisao.washington.edu/pdo) computed as the principal component of monthly SST anomalies in the specific domain (poleward of 20°N, from 20°–70°N, 110°E–100°W). We also used the global SST data, obtained from the Program for Climate Model Diagnosis and Intercomparison (https://pcmdi.llnl.gov/mips/amip/amip2) to identify the influence on the extremes of the Pw.

### Methodology

By referring to the definition and calculation method described elsewhere^20,21^, the Pw for an irregular sea-state was derived from the wave spectral parameters using Eq. (1):

1 $${P}_{w}=\frac{\rho {g}^{2}}{64\phi }\times {T}_{p}\times {({H}_{s})}^{2}$$

where *ρ* is the water density, *g* is the gravitational acceleration with 9.8 m/s, *T*_*p*_ is the peak wave period, and *H*_*s*_ is the average of the highest third of the waves in a time-series of waves. Eq. (1) estimates the Pw, which increases nonlinearly with *H*_*s*_ and is proportional to *T*_*p*_. The *T*_*p*_ is sensible to the wavelength and duration of the wave fetch over which strong winds persist. Estimating *P*_*w*_ helps us to consider the *T*_*p*_ as well as the *H*_*s*_ because the effects of storms on coastal processes depend on the wave power energy that is propagated by the waves. Therefore, contrary to *H*_*s*_ alone, the Pw provides useful information on wave effects. The summer wave parameters (i.e., Hs, Tp, and Pw) in the WNP covering [20°S–70°N, 90°E–150°W] were then calculated from the wave data set at three-hourly intervals. The analysis domain includes parts of the Southern Hemisphere and ENP. Moreover, the 99^*th*^ percentile of wave parameters were defined to be the extremes of the wave parameters (e.g., Hs_99_). The spatial distribution of climatological averaged wave parameters was computed to identify the regional characteristics during JJA.

We examined the responses of SST, SLP, and wave parameters on the ENSO and the PDO from 1979 to 2009 during JJA by using the linear regression method. In the regression analysis, all variables were standardized by the distribution mean and standard deviation for each variable as $x{\prime} =(x-\mu )$/*σ*, where x is the original variable, ${x}^{{\prime} }$ is the standardized variable, *μ* is the mean of that variable, and *σ* is its standard deviation. For SLP, the unit of measurement of the slope of regression is hPa (standard deviation)^−1^, i.e., no units. Furthermore, to investigate the combined influences of the dominant climate mode in the WNP during JJA, we conducted a composite analysis of the SST, SLP, and Pw_99_ anomalies on different phase combinations of the ENSO and PDO. All anomalies are computed relative to the 31-year period from 1979–2009. To obtain composite samples, we selected several years from +ENSO and −ENSO based on the ONI indices, and +PDO and −PDO for comparison to the standard deviations. We also evaluated the relationship between the Pw_99_ anomalies and the TS activities representing the wind-speed energy in the WNP during JJA by considering the averages for each composite. In particular, a detecting method of TS in Murakami and Sugi (2010)^43^ was used to detect candidates of TS from 3-hourly raw data in the ICON model. The TWI is derived as latitude-weighted averages of zonal wind anomalies at 850 hPa over the western central Pacific (135°E–180°E, 5°S–5°N), where the positive (negative) values of the TWI indices imply easterly (westerly) anomalies. All atmospheric data sets used in the present study were obtained from the ICON model of Yang and Oh (2017)^1^.

## Supplementary information

Supplementary Information.

## Data Availability

The ONI index is obtained from the Climate Prediction Center (CPC)’s website (https://www.cpc.ncep.noaa.gov/data/indices/) of the National Oceanic and Atmospheric Administration (NOAA). The PDO index is also downloaded from the Joint Institute for the Study of the Atmosphere and Ocean (JISAO)’s website (http://research.jisao.washington.edu/pdo/PDO.latest). The global SST data for 1979–2009 is obtained from the website (https://pcmdi.llnl.gov/mips/amip/amip2).
