# Supplementary material for: Effects of modes of climate variability on wave power during boreal summer in the western North Pacific
Source: Sci Rep. 2020 Mar 20;10:5187. doi: 10.1038/s41598-020-62138-0 (PMC7083846; doi:10.1038/s41598-020-62138-0)
Supplement: Supplementary file 1 — Supplementary Information. [file 41598_2020_62138_MOESM1_ESM.docx]

Supplementary Information for

**Effects of modes of climate variability on wave power during boreal summer in the western North Pacific**

Sinil Yang^1^ and Jai-Ho Oh^2^

^1^ Korea Ocean Satellite Center, Korea Institute of Ocean Science and Technology, Busan, 49111, Republic of Korea

^2^ Department of Environmental Atmospheric Sciences, Pukyong National University, Busan, 48513, Republic of Korea

**Corresponding authors**: Jai-Ho Oh: [jhoh@pknu.ac.kr](mailto:jhoh@pknu.ac.kr)

**This file includes**:

Supplementary Text S1 and S2

Supplementary Figures S1 and S2

References for SI reference citations

# **Supplementary Text S1:** Description of the ICON model

Zonal and meridional wind components above 10-meter (i.e., sea surface wind fields) are simulated to reproduce wave characteristics in a spectral wave model for the 31 years from an atmospheric general circulation model, global Icosahedral Non-hydrostatic model (hereafter ICON). ICON is a joint development project of the Max Planck Institute for Meteorology (MPI-M) and Germany's National Meteorological Service (Deutscher Wetterdienst, DWD), and also a unified modeling system for global climate modeling and numerical weather prediction (NWP) (Zängl et al., 2015). A non-hydrostatic dynamical core of ICON, one of the essential features of ICON, allows the single model to be suitable for seamless prediction in various temporal ranges of spatial resolutions. Also, the discretization of the primitive equations of ICON is implemented on an icosahedral-triangular Arakawa C-grid discretization (Arakawa and Lamb, 1977). In particular, ICON has high efficiency and scalability on future massively parallel high-performance computing architectures and solves the fully compressible nonhydrostatic atmospheric equations of motion, using the set of prognostic variables suggested by Gassmann and Herzog (2008). Atmospheric variables then are sequentially updated with physical parameterizations (e.g., cloud microphysics, turbulence, and radiation). Here, the parameterizations have been partly imported from the Consortium for Small-scale Modelling (COSMO) model, which is a non-hydrostatic limited-area atmospheric prediction model, and partly from the Integrated Forecast System (IFS). From the COSMO model, we inherited the five category cloud microphysics scheme that is operational in the COSMO-EU (Schättler et al., 2008; Seifert, 2008), the turbulence kinetic energy (TKE) based turbulence scheme (Raschendorfer, 2001) and the TERRA land-surface scheme (Heise et al., 2006). From the IFS, we imported the moist convection scheme (Bechtold et al., 2008), the subgrid-scale orography scheme (Lott and Miller, 1997), and the non-orographic gravity-wave drag scheme (Orr et al., 2010). Moreover, the Rapid Radiative Transfer Model (RRTM) radiation scheme (Mlawer et al., 1997) is used in a similar version as in the IFS.

# **Supplementary Text S2:** Validation for the simulated wave parameters

A gridded hindcast allows for an assessment of wave parameters from simulation outputs, which is a 30-year wave hindcast of the National Centers for Environmental Prediction (NCEP) Climate Forecast System Reanalysis (CFSR). This latest wave hindcast (hereafter denoted as CFSR) provides 3-hourly global data with a spatial resolution of 0.5°, which is considered a reliable dataset among the global hindcasts. CFSR hindcasts can be downloaded free of charge from a website (<https://polar.ncep.noaa.gov/waves/hindcasts/nopp-phase2/>). In the present study, a gridded monthly mean of significant wave heights (Hs) and peak wave period (Tp) is validated with CFSR hindcast. Here, we have conducted to evaluate monthly Hs and Tp between CFSR and simulation (see Figure S1).

Figure S1 shows a temporal correlation of the wave parameters between simulation and CFSR in the analysis domain (90°E to 150°W longitude, 20°S to 70°N latitude). A two-tailed Student’s t-test is used for a significance test of the correlation coefficient $\text{r}$ at a confidence level of 0.1% with degrees of freedom n=124. Black dots also represent grid points with a statistically significant correlation in Fig. S1. In this study, simulation results in each grid point are spatially interpolated into a resolution of 0.5° in a regular grid, due to a coarser spatial resolution of CFSR hindcast. First, simulation results were well simulated for the inter-monthly variability of both Hs and Tp in the North Pacific (Fig. S1). Here, we obtained the most significant correlation (p<0.001) in the central North Pacific (CNP), and this significant correlation mostly corresponds to the overall analysis domain. In particular, the correlation of open oceans (e.g., the mid-latitudes of the North Pacific) is higher than in coastal areas (e.g., the western coast of the WNP and the Southern Ocean) in Fig. S1a. Similarly, modeled Tp has a statistically positive correlation in the open oceans of the North Pacific except for several coastal regions (i.e., no significant in parts of the Okhotsk Sea and the Bering Sea) in Fig. S1b. Consequently, the simulation result is in good agreement with the CFSR hindcast. The simulation results, therefore, are useful in the analysis domain and allows us to employ climate research.

# **Supplementary Figures**


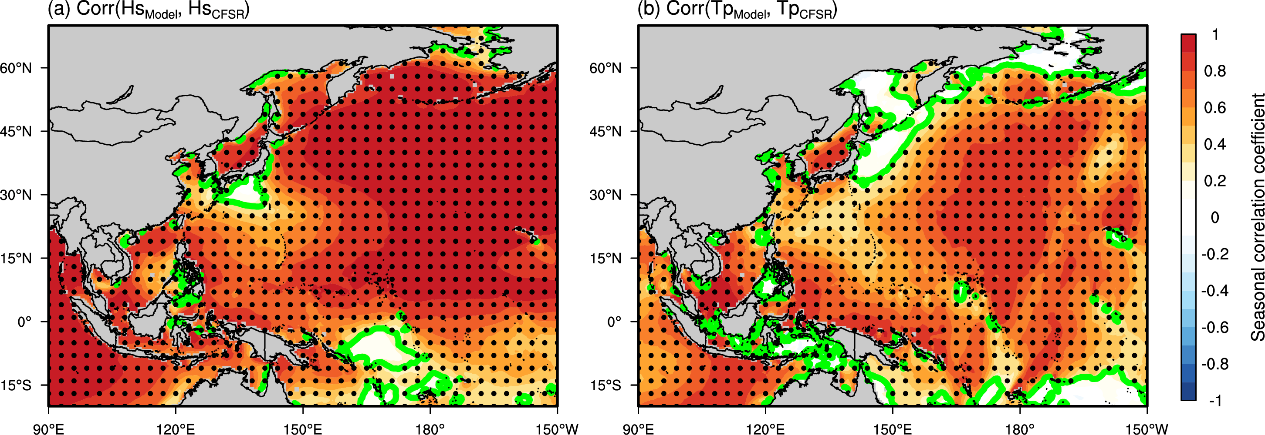


**Figure S1.** Comparison of correlation coefficient of (left) Hs and (right) Tp between simulation output and CFSR hindcast. A two-tailed Student’s t-test is used for the significance test of the correlation coefficient. Black dots denote a significant correlation at a confidence level of 0.1% (p<0.001 at n=124).


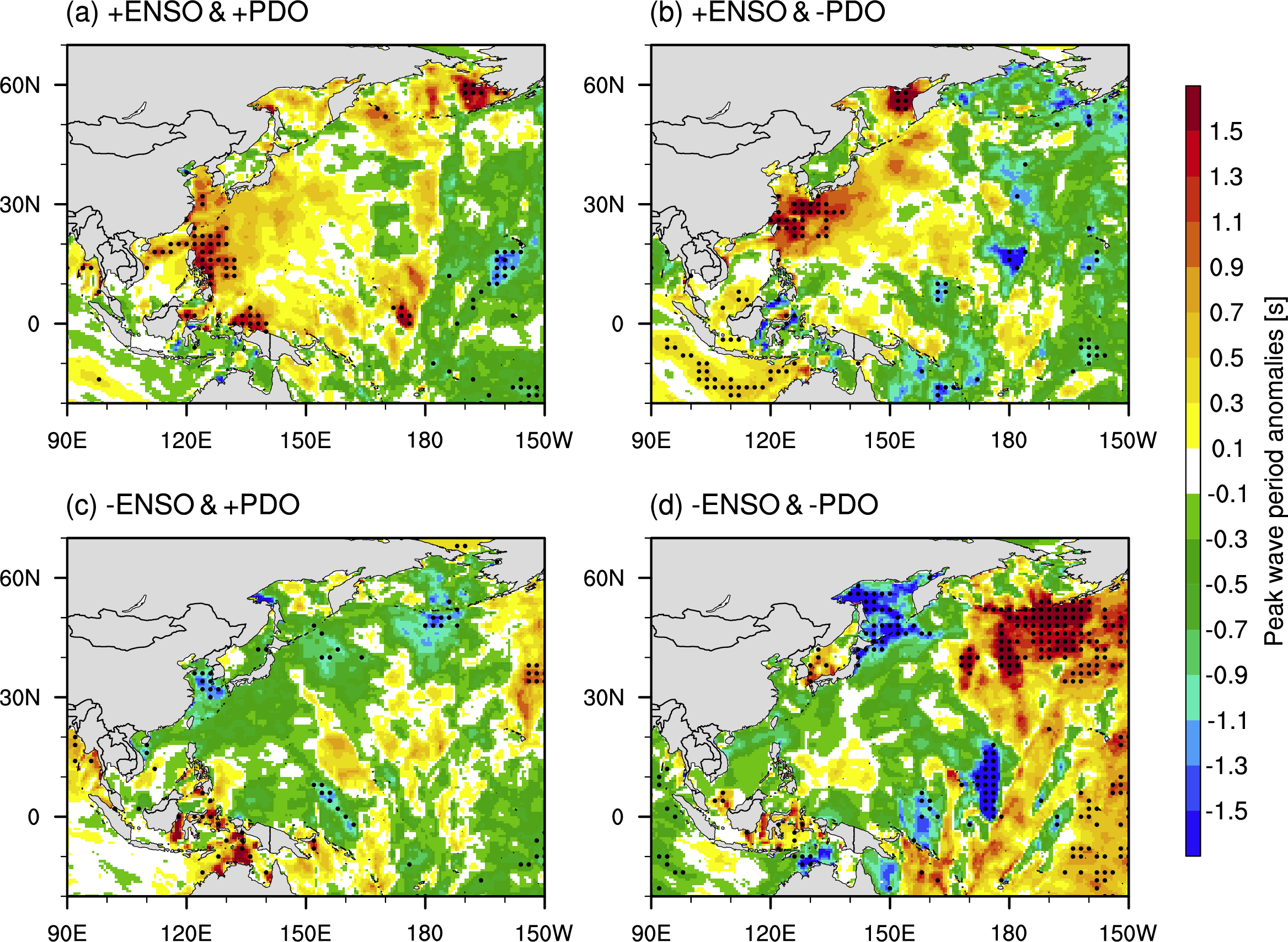


**Figure S2**. Composite patterns of Tp_99_ [shaded, in s] anomalies for the four combinations of the El Niño-Southern Oscillation (ENSO) and the Pacific Decadal Oscillation (PDO), which are the (a) El Niño (+ENSO)/+PDO, (b) +ENSO/-PDO, (c) La Niña (-ENSO)/+PDO, and (d) -ENSO/-PDO years. Anomalies are computed relative to the 31 years from 1979–2009. Black dots denote the significant Tp anomalies at a confidence level of 90%.

# **Supplementary references:**

Arakawa, A. & Lamb, V.R. Computational design of the basic dynamical processes of the UCLA general circulation model. *Methods of Computational Physics*, **17**, 173–265, DOI: https://10.1016/B978-0-12-460817-7.50009-4 (1977).

Bechtold, P. *et al.* Advances in simulating atmospheric variability with the ECMWF model: From synoptic to decadal time-scales. *Quarterly Journal of the Royal Meteorological Society*, **134(634)**, 1337–1351, DOI: https://10.1002/qj.289 (2008).

Gassmann, A. & Herzog, H. J. Towards a consistent numerical compressible non-hydrostatic model using generalized Hamiltonian tools. *Quarterly Journal of the Royal Meteorological Society*, **134(635)**, 1597–1613, DOI: https://10.1002/qj.297 (2008).

Heise, E., Ritter, B. & Schrodin, E. Operational implementation of the multilayer soil model TERRA. Technical report. Deutscher Wetterdienst: Offenbach, Germany. http://www.cosmo-model.org (2006).

Lott, F. & Miller, M. J. A new subgrid-scale orographic drag parametrization: Its formulation and testing. *Quarterly Journal of the Royal Meteorological Society*, **123(537)**, 101–127, DOI: https://10.1256/smsqj.53703 (1997).

Mlawer, E. J., Taubman, S. J., Brown, P. D., Iacono, M. J. & Clough, S. A. Radiative transfer for inhomogeneous atmospheres: RRTM, a validated correlated-k model for the longwave. *Journal of Geophysical Research: Atmospheres*, **102**, 16663–16682, DOI: https://10.1029/97JD00237 (1997).

Orr, A., Bechtold, P., Scinocca, J., Ern, M. & Janiskova, M. Improved middle atmosphere climate and forecasts in the ECMWF model through a nonorographic gravity wave drag parameterization. *Journal of Climate*, **23(22)**, 5905–5926, DOI: https://10.1175/2010JCLI3490.1 (2010).

Raschendorfer, M. The new turbulence parameterization of LM. *COSMO newsletter*, **1**, 89–97 (2001).

Schättler, U., Doms, G. & Schraff, C. A description of the nonhydrostatic regional COSMO-model part VII: user’s guide. Deutscher Wetterdienst Rep. COSMO-Model, **4**, 142 (2008).

Zängl, G., Reinert, D., Rípodas, P. & Baldauf, M. The ICON (ICOsahedral Non-hydrostatic) modelling framework of DWD and MPI-M: Description of the non-hydrostatic dynamical core. *Quarterly Journal of the Royal Meteorological Society*, **141**, 563–579, DOI: https://10.1002/qj.2378 (2015).
